# Rotation is visualisation, 3D is 2D: using a novel measure to investigate the genetics of spatial ability

**DOI:** 10.1038/srep30545

**Published:** 2016-08-01

**Authors:** Nicholas G. Shakeshaft, Kaili Rimfeld, Kerry L. Schofield, Saskia Selzam, Margherita Malanchini, Maja Rodic, Yulia Kovas, Robert Plomin

**Affiliations:** 1 Medical Research Council Social, Genetic and Developmental Psychiatry Centre, Institute of Psychiatry, Psychology & Neuroscience, King’s College London, London, United Kingdom; 2 Goldsmiths, University of London, New Cross, London, United Kingdom; 3 University of Sussex, Sussex House, Falmer, Brighton, United Kingdom; 4 Tomsk State University, Tomsk, Russia

## Abstract

Spatial abilities–defined broadly as the capacity to manipulate mental representations of objects and the relations between them–have been studied widely, but with little agreement reached concerning their nature or structure. Two major putative spatial abilities are “mental rotation” (rotating mental models) and “visualisation” (complex manipulations, such as identifying objects from incomplete information), but inconsistent findings have been presented regarding their relationship to one another. Similarly inconsistent findings have been reported for the relationship between two- and three-dimensional stimuli. Behavioural genetic methods offer a largely untapped means to investigate such relationships. 1,265 twin pairs from the Twins Early Development Study completed the novel “Bricks” test battery, designed to tap these abilities in isolation. The results suggest substantial genetic influence unique to spatial ability as a whole, but indicate that dissociations between the more specific constructs (rotation and visualisation, in 2D and 3D) disappear when tested under identical conditions: they are highly correlated phenotypically, perfectly correlated genetically (indicating that the same genetic influences underpin performance), and are related similarly to other abilities. This has important implications for the structure of spatial ability, suggesting that the proliferation of apparent sub-domains may sometimes reflect idiosyncratic tasks rather than meaningful dissociations.

Spatial ability is one of the most widely-studied domains of cognitive ability, yet there is little consensus as to its nature or structure. It has been found to be a strong predictor of important outcomes, such as science, technology, engineering and maths (STEM) performance^1^, but its usefulness in this regard is limited by the lack of understanding about its basic architecture. Broadly defined, the spatial domain comprises the processes involved in perceiving, memorising and manipulating mental representations of visual scenes^2^, including two-dimensional (2D) and three-dimensional (3D) objects^1^,^3^ and the relationships between them^4^. Putative processes, categories and sub-domains–such as visualisation^5^, spatial orientation^6^, mental rotation^7^, spatial relations^6^ and many others–have proliferated in the literature, often with overlapping definitions, to the extent that the term “spatial ability” itself is difficult even to define with precision^8^,^9^.

A great many spatial tests have been developed and are commonly used, with varying intercorrelations among them, and several theories have been proposed to describe the multifactorial structure suggested by these relationships^4^,^9^,^10^. Two major putative sub-domains (among many others) are “mental rotation” and “visualisation”. Definitions vary, but mental rotation involves rotating mental models of objects into different orientations, and visualisation describes various complex mental manipulations of spatial information, including identifying hidden or partially occluded objects from incomplete information^11^. Theories differ as to the nature of these abilities and the relationship between them, with some proposing that they represent distinct sub-domains of spatial ability^5^, while others suggest that visualisation is a major sub-domain, of which mental rotation is merely a component or exemplar^9^. Similarly, investigating the effects of the dimensionality of stimuli has led to contradictory results, with some studies^3^,^12^ finding differences between the processing of 2D and 3D stimuli, and other results^9^,^13^ suggesting otherwise. One possible explanation for some of the inconsistent findings in the literature is that the available tests may not be “pure”, in the sense that their items may conflate multiple cognitive processes such that factor analyses cannot distinguish them^4^. Another possibility, primarily concerning the apparent distinction between 2D and 3D stimuli, is that test items may differ substantially in complexity^3^.

Behavioural genetic methods may provide a different perspective, as yet largely unexplored, from which to clarify the nature of spatial abilities and the aetiology of their interrelationships. These methods concern individual differences, rather than the normative focus of much cognitive work. Several studies have observed substantial familiality (i.e., resemblance among related individuals) for spatial abilities^14^,^15^,^16^,^17^. Adoption^18^ and twin^19^,^20^,^21^ studies have found this familiality to be substantially genetic in origin, with average heritability estimates at around 50% for spatial ability in adulthood. However, for the purpose of elucidating the structure of individual differences within and between domains, multivariate genetic analyses–permitting calculation of the genetic and environmental influences shared between multiple observed traits^22^–are more informative: if two traits are meaningfully and fundamentally dissociable (in their neurobiological basis, for example), we might reasonably predict this to be reflected in their genetic aetiology. Such methods have been applied to investigate the degree to which spatial ability shares common genetic influences with other cognitive domains such as mathematical ability^23^, finding a moderate overlap. However, to date no multivariate genetic studies have been published examining the genetic architecture *within* the spatial domain itself.

Thus the present study had two main aims. First, a novel battery of spatial tests was developed and validated with the express purpose of allowing i) mental rotation and ii) visualisation *without* rotation (e.g., picturing a whole object from incomplete information) to be tested in isolation from one another, using both 2D and 3D stimuli of approximately equivalent complexity. In this way, the relationship between mental rotation and visualisation, and between 2D and 3D stimuli, could be examined without confounds. Second, this new battery was administered to a large twin sample, together with other cognitive measures, in order to assess the extent to which any dissociation between these different types of stimuli may be attributed to genetic or environmental factors.

## Results

### Data

The Twins Early Development Study (TEDS) is a longitudinal cohort study of more than 10,000 pairs of British twins, born between 1994 and 1996. The sample is representative of the population of the United Kingdom, and has been described previously^24^. For the present study, a representative subsample was selected from among the older twins in the cohort, who had completed a battery of cognitive tests on a previous occasion (at age 16), assessing their verbal ability (with the Mill Hill Vocabulary Scale^25^) and non-verbal ability (Raven’s Progressive Matrices^26^), from which a proxy of their general cognitive ability (*g*) could be derived as the mean of these two standardised scores.

**Figure 1:**
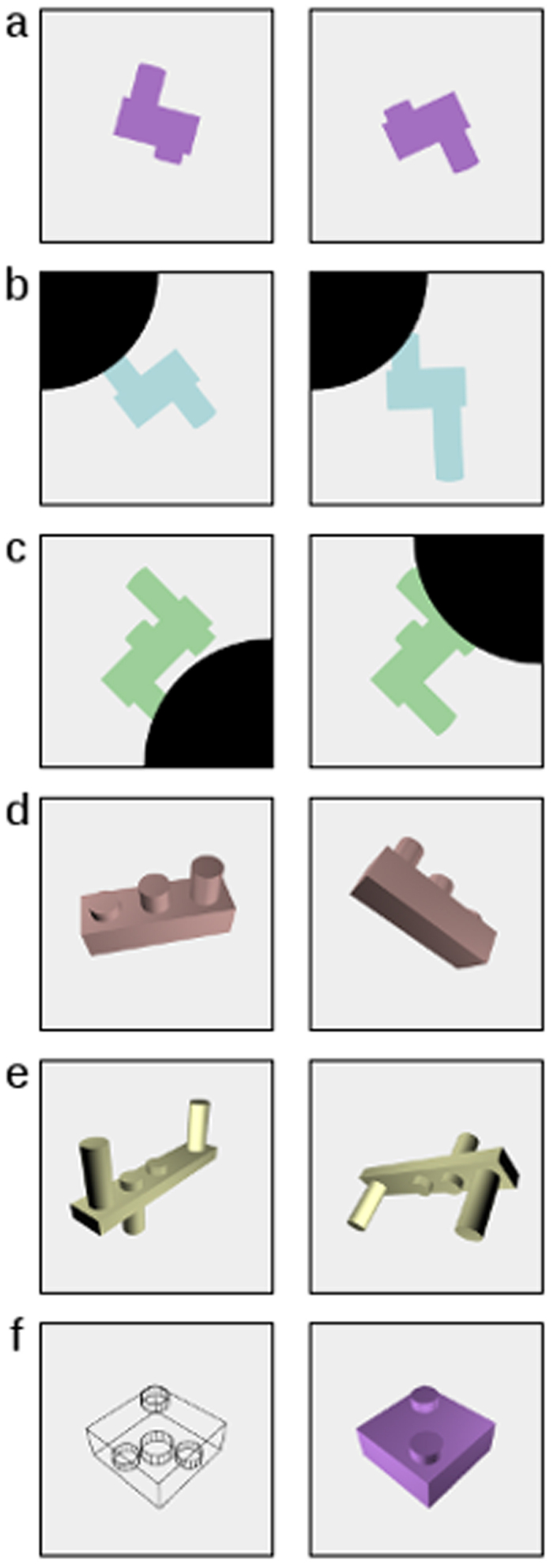
Sample stimuli. Sample target images (left) and correct responses (right) for the six Bricks subtests: (**a**) 2D Rotation, (**b**) 2D Rotation/Visualisation combined, (**c**) 2D Visualisation, (**d**) 3D Rotation/Visualisation combined, (**e**) 3D Rotation, and (**f**) 3D Visualisation.

This TEDS subsample was asked to complete a novel battery of spatial tests: the “Bricks” battery. This consisted of six subtests, assessing either mental rotation alone, spatial visualisation alone (without rotation), or both together, using either two- or three-dimensional stimuli. Three “functional” composites (“Rotation”, “Visualisation”, and “Rotation/Visualisation combined”, each being the mean of the 2D and 3D subtests of that type), and two “dimensional” composites (“2D” and “3D”, each being the mean of the three corresponding subtests) were derived from these subtest scores. As a marker of overall spatial ability (for reference), an “Overall Bricks” composite was also derived as the mean of all six subtest scores. Details are presented in Methods, with examples of the stimuli in Fig. 1. These stimuli were prepared using purpose-built software allowing computer-generated objects to be manipulated dynamically; this software is freely available here: https://www.forepsyte.com/resources/public

The data were cleaned and prepared as described in Methods. The final dataset comprised 2,913 participants: 1,250 twin pairs (528 monozygotic (MZ), 722 dizygotic (DZ)), and an additional 413 unpaired individuals (104 from MZ and 309 from DZ pairs). The participants were 63% female (the gender imbalance reflecting a disparity in response rates), with a mean age of 20.3 years (±0.47 SD) on completing the Bricks tests.

Sample sizes and descriptive statistics for the Bricks subtests and composites, and the other measures, are presented in Supplementary Table S1. The reliability of the Bricks battery was assessed with regard to Cronbach’s alphas, and also test-retest correlations in an independent pilot sample. Bricks composite alphas ranged from 0.63 to 0.85, and test-retest correlations from 0.62 to 0.83; for details, see Supplementary Table S2.

For each measure, an analysis of variance assessed the mean effects of sex and zygosity (Supplementary Table S1). As is often observed for spatial abilities^27^, a main effect of sex was found for all Bricks measures, representing a slight male advantage (average R^2^ = 0.03 for the Bricks composites). Mean sex differences are irrelevant to twin analyses, which examine variances, but common practice for twin studies is to analyse sex- and age-corrected residuals (see Methods). For all subsequent analyses, the data were regressed on age and sex, normality-transformed and standardised.

### Phenotypic analyses

For all phenotypic analyses, one twin was selected at random per pair to create an independent sample.

Preliminary analyses suggested immediately that the putative distinctions in some of the literature between mental rotation and spatial visualisation, and between 2D and 3D stimuli, were not supported. The modest intercorrelations among the six subtest scores (*r* ranging from 0.25 to 0.42; see Supplementary Table S3) revealed no apparent clusters of stronger or weaker associations. For example, the 2D subtests showed no consistently stronger correlations with one another than with the 3D subtests, nor were the Rotation subtests associated more substantially with each other than with the Visualisation subtests. To examine this more formally, the subtest scores were subjected to factor analysis, producing only a single factor on which all six subtests were strongly loaded (with factor loadings ranging from 0.57 to 0.70; see Supplementary Table S4).

However, it must be noted that the subtests were not intended for use in this way, being very short individually in comparison to most cognitive tests–and thus not very highly reliable–in order to keep the administration of the whole battery within a reasonable time limit. The results from the individual subtests should therefore be treated with caution, and the Bricks composites were created on the original theoretical grounds, to assess whether clearer distinctions might emerge from the more reliable constructs.

The resulting functional composites were moderately intercorrelated. If mental rotation and spatial visualisation are functionally distinct, we would predict the Rotation and Visualisation composites to be correlated more modestly with each other than either is with Rotation/Visualisation combined. In fact, the results showed that the association between Rotation and Visualisation (*r* = 0.46, p < 0.0001, N = 1411) was identical to that between Rotation and Rotation/Visualisation combined (*r* = 0.46, p < 0.0001, N = 1423), and the correlation between Visualisation and Rotation/Visualisation combined (*r* = 0.54, p < 0.0001, N = 1426; the slight variations in sample size result from losses during data cleaning, described in the Supplementary Methods online) did not differ substantially (although the small difference was significant in this large sample; p < 0.001). However, these correlations are far from unity, as is that between the 2D and 3D composites (*r* = 0.56, p < 0.0001, N = 1413), which suggests some specificity between the composites. The nature of this specificity is the subject of the multivariate genetic analyses below.

The Bricks composites correlated modestly with verbal ability (average *r* = 0.20), and moderately with non-verbal ability (*r* = 0.43) and *g* (*r* = 0.38); see Supplementary Table S5. It was considered that the associations among the Bricks scores could be driven in part by more domain-general abilities or processes captured by these other measures, which could potentially obscure the “true” relationships among the Bricks subtests and composites. Accordingly, the Bricks subtests and composites were regressed separately on verbal ability (a conservative under-correction for domain-general processes; see Methods), on non-verbal ability (perhaps an over-correction including some of the variance in spatial ability, reflected in its higher correlations with Bricks), and on *g* (their mean). The strength of the relationships among the resulting subtest and composite residuals was reduced slightly and uniformly, with no different patterns emerging among either the subtests (see Supplementary Tables S6–S8) or composites (Supplementary Tables S9–S12). The factor analysis results were similarly unaffected (Supplementary Table S13), implying that *g* does not mask differentiation among the spatial subtests.

### Univariate genetic analyses

Intraclass twin correlations are presented in Table 1 for the Bricks composites, and in Supplementary Table S14 for the Bricks subtests and other cognitive measures. These intraclass correlations may be used to calculate initial estimates for the “heritability” (additive genetic influences), “shared environment” (environmental factors promoting similarity) and “non-shared” or “unique environment” (environmental factors not contributing to similarity between twins, and also any measurement error) influencing the trait–see Table 1 for details. The resulting estimates (Table 1) indicate substantial genetic influence on all measures, up to 56% for the Overall Bricks composite.

To establish these estimates more precisely, and to obtain model fit statistics and confidence intervals (CIs), the data for each measure were subjected to maximum-likelihood model-fitting to estimate the portions of variance attributable to additive genetic (A), shared environmental (C) and non-shared (unique) environmental components (E, also including measurement error). See Methods for details. The results confirm that all Bricks composites are moderately heritable (Table 2), with no significant differences in the magnitude of the genetic influences between the various functional composites, or between the two dimensional composites. There were substantial non-shared, but no significant shared environmental influences. Results for the individual Bricks subtests and other cognitive measures are presented for reference in Supplementary Table S15.

### Multivariate genetic analyses

**Figure 2:**
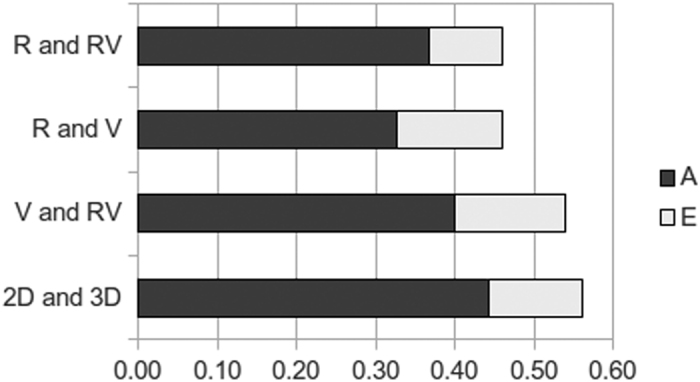
Decomposition of phenotypic correlations. Correlated factor solution analyses, indicating the proportion of the phenotypic correlations (line length) among the Bricks composites attributable to genetic (**A**) shared environmental (**C**) and non-shared environmental influences/error (**E**). R = Rotation, RV = Rotation/Visualisation combined, V = Visualisation.

Bivariate correlated factors solutions (see Methods) were fitted to each pair of Bricks composites in turn, from which their phenotypic correlations could be decomposed into the proportions attributable to genetic, shared and non-shared environmental influences. The results (Fig. 2, with precise estimates and CIs in Supplementary Table S16) indicate that the phenotypic correlations are largely (70–80%) genetic in origin, with the remainder due to non-shared environmental influences. Similar patterns appear between the individual subtests (Supplementary Tables S17 and S18). The correlations between the Bricks composites and the other cognitive measures are also substantially genetically driven, with shared genetic influences accounting for approximately all of the relationships with verbal ability, and a majority (64% on average) of the stronger relationships with non-verbal ability (Supplementary Table S19).

**Figure 3:**
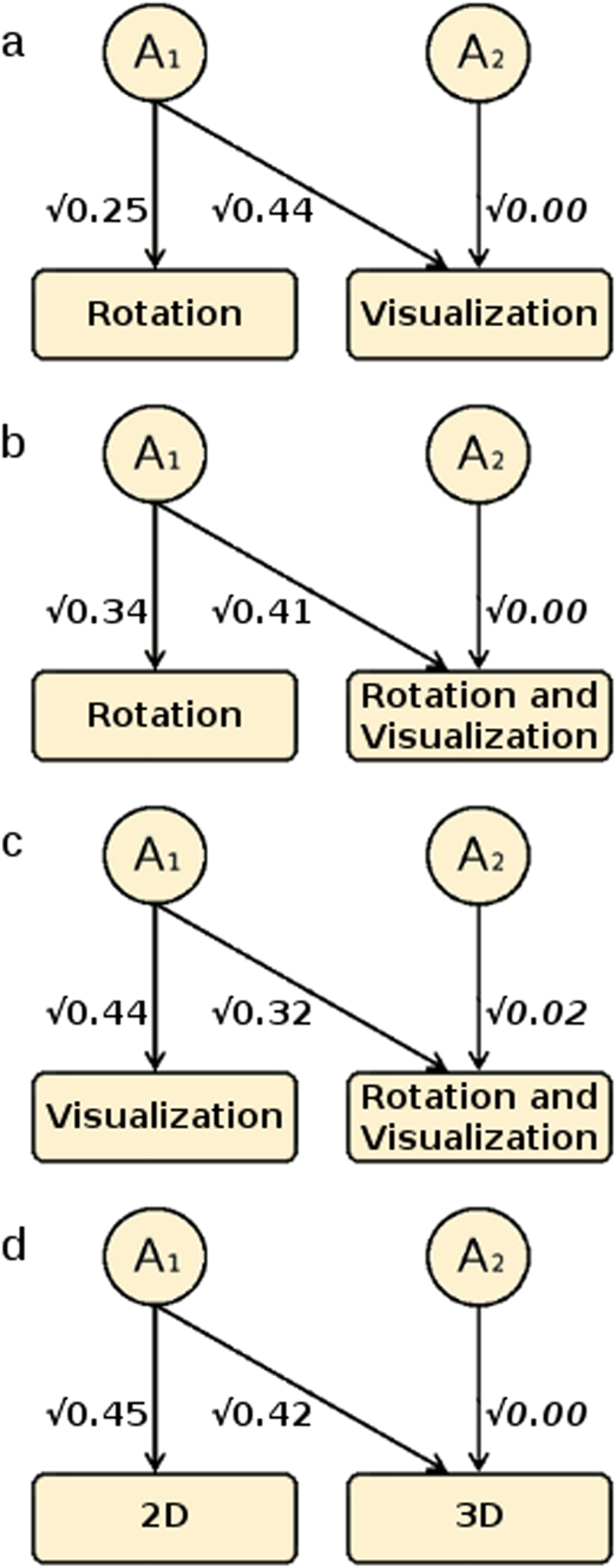
Decomposition of heritability. Four bivariate Cholesky decompositions indicating the genetic relationship between (**a**) Rotation and Visualisation, (**b**) Rotation and Rotation/Visualisation combined, (**c**) Visualisation and Rotation/Visualisation combined, and (**d**) 2D and 3D. Independent paths (italicised) are all non-significant.

As these results only decompose the phenotypic correlations, they do not directly estimate the portions of variance that are unique to each variable–that is, they do not reveal what proportions of the *total* influences on each composite are shared with others. This is the purpose of Cholesky decomposition (Methods). These results (Fig. 3 and Supplementary Tables S20–S23) suggest, for each bivariate relationship among the Bricks composites, that 100% of the substantial genetic influences on each composite measure is shared with all the others. This can be seen in Fig. 3: in each model, all of the genetic variance of the second variable (on the right) is shared with the first, resulting in a loading of 0 for the residual genetic path for the second variable.

This pattern is revealed even more starkly by the genetic correlations, which indicate the correlation between genetic influences on the two variables independent of their heritabilities (Methods). These are all at unity among the Bricks composites (Supplementary Tables S24 and S25). Even for the comparatively unreliable individual subtests, the genetic correlations are all either at unity or have CIs including unity (Supplementary Table S26).

As there are no significant shared environmental influences on any of the Bricks measures, there are no meaningful correlations between these components. However, the correlations between *non*-shared environmental influences (Supplementary Tables S24, S25 and S27) indicate that there are modest “unique” environmental effects in common between the measures (i.e., effects unique to each individual, but affecting multiple traits), up to a maximum rE = 0.23 between Bricks composites.

The genetic correlations between the Bricks composites and the other cognitive measures (Supplementary Table S28) indicate a substantial genetic overlap (average rA = 0.55) with verbal ability, higher still with non-verbal ability (average rA = 0.71), and the association with *g* (their mean) unsurprisingly in between (average rA = 0.65).

As with the phenotypic results, it was considered that the genetic associations among the Bricks measures could reflect domain-general influences shared with other cognitive abilities, too, rather than influences specific to spatial abilities. Multivariate Cholesky decompositions (see Methods) were performed for Rotation and Visualisation, and for 2D and 3D, first accounting for the genetic influences on verbal ability, non-verbal ability, or both, and then examining the residual relationships between the Bricks composites. In these trivariate models, verbal ability accounts for less than one third of the heritability of the Bricks composites, non-verbal ability for around half (but the difference is non-significant), and *g* (their mean) in between. In two quadrivariate models (entering verbal and non-verbal ability separately, then Rotation and Visualisation or 2D and 3D), the verbal and non-verbal cognitive measures accounted in total for around half of the heritability of the Bricks measures. In every model, substantial genetic influence remains that is unique to spatial ability as a whole, supporting it as a distinct cognitive domain from *g.* However, none of the genetic variance is unique to any specific Bricks composite–all genetic influences are shared between all Bricks measures.

Detailed results are presented in the Supplementary Materials online: Supplementary Figs S1 and S2 for illustration, and full details in Supplementary Tables S29–S36. Fit statistics for the Bricks composite models are presented in Supplementary Tables S37–S40.

## Discussion

The Bricks battery was designed with the express purpose of differentiating between mental rotation and spatial visualisation, and to assess them equally in 2D and in 3D. The key multivariate genetic results all show a strong and consistent pattern, for the functional composites (Rotation, Visualisation, Rotation/Visualisation combined), dimensional composites (2D, 3D), and even for the individual subtests: it is impossible, genetically at least, to distinguish between any of these spatial constructs (Fig. 3). Once the genetic influences on any one of these measures are accounted for, nothing remains. As specific genes are identified that are associated with any of these spatial abilities, it is expected that these genes will be similarly associated with all of them.

Phenotypically, the results arguably present a more ambiguous picture, since the intercorrelations are modest among the Bricks subtests, and moderate (average *r* = 0.51) even among the more reliable composites. There are many reasons why phenotypic correlations might be imperfect, of course, without this reflecting theoretically meaningful dissociations–there can be unintended test-specific differences, for example. However, the most likely explanation in this instance is reliability: the test-retest correlations for the Bricks composites are respectable but far from unity (average *r* = 0.69), so the measures do share a large majority of their *reliable* phenotypic variance (i.e., 74% overall). In any case, the other phenotypic results show no evidence of any dissociations: factor analysis produces only a single factor with no substantial differences in loadings between the subtests, and the Bricks measures all present very similar patterns of correlations with the other cognitive measures assessed. Taken together, there is no more evidence of meaningful dissociations phenotypically than genetically.

While the genetic associations between the Bricks measures account for a majority of the phenotypic correlations between them (Fig. 2), a significant minority is driven by modest correlations between their non-shared environmental influences (Supplementary Tables S24, S25 and S27); i.e., E in the ACE models (Methods). These are environmental influences unique to each participant, making co-twins less similar to one another, but which influence multiple traits and increase their correlations–these could be personal traits affecting performance across multiple tests, or indeed situational factors such as the participant’s testing environment. This non-shared component is the only source of environmental influence common to multiple Bricks measures, and the absence of any significant *shared* environmental influences (i.e., C in the ACE models) is striking. For the Bricks measures and everything they capture, genetic influences are the only source of familial similarity.

The tests were developed specifically to differentiate cleanly between mental rotation and spatial visualisation. The lack of any genetic (or even any unambiguous phenotypic) specificity between the Rotation and Visualisation composites would seem to provide strong support, therefore, for the previous literature^9^ suggesting that they do not represent meaningfully dissociable tasks, and to refute the suggestions^5^ to the contrary. While we cannot draw any conclusions about the specific mechanisms of action of any influences, it also suggests an absence of distinguishable cognitive processes underlying them. Stated more boldly, mental rotation is nothing more than visualisation, and likewise visualisation recruits no distinct processes even when rotation is not required. Where differentiation has been observed previously in this area, it seems plausible that this reflects task-specific effects or reliability issues, rather than theoretically meaningful differences.

Some of the previous reports of dissociation between 2D and 3D stimuli suggested that the difference might relate to 3D objects being more complex, and therefore more time being required to encode their mental representations^3^. While response times were not included directly in the Bricks scores reported here, the 2D and 3D Bricks composites were intended to be approximately equal in difficulty, and the inclusion of restrictive item time limits (see the Supplementary Methods online) would have been expected to affect scores if the 3D items had been substantially harder than the 2D items; there is no evidence of this (indeed the 3D mean score is marginally higher than 2D; Supplementary Table S1). This suggests that the 2D and 3D Bricks composites are indeed of broadly equivalent difficulty. Coupled with the clear lack of differentiation between these composites in the results, this supports the contention that differences in difficulty–rather than fundamental differences in the processes involved–are responsible for the dissociations sometimes observed.

It must be emphasised that there are a great many putative sub-domains of spatial ability not included in the present study. Likewise, even the definition of “visualisation” used here is quite narrow–definitions vary in the literature, but visualisation is sometimes taken to include more complex mental manipulations than those operationalised in the Bricks measures. The present results should not be over-interpreted beyond the abilities assessed, therefore, but it is hoped that they may indicate a fruitful approach. In subsequent work, we will apply these methods to more diverse abilities sampled from across the spatial domain.

The importance of spatial ability for outcomes such as STEM performance^1^ is well documented, and it is to be hoped that clarifying the nature and structure of this domain will refine its measurement and increase its utility further. It should be noted that, while no differentiation *within* the spatial domain was supported by these results, the correlations between the Bricks measures and the other cognitive measures examined were only moderate, both phenotypically and genetically (Supplementary Tables S5 and S28), despite the probable inclusion of some spatial elements within the non-verbal cognitive measure itself (Methods). This certainly supports the existence of spatial ability as a distinct cognitive domain in its own right.

As noted above, the structure of this distinct spatial domain is hotly contested, and seemingly always growing in its apparent size and complexity. Where previous findings have suggested meaningful dissociations between visualisation and mental rotation, though, and between 2D and 3D stimuli, the present study suggests that it is possible to shrink it, too.

## Methods

### Measures

The Bricks battery comprises six subtests of nine items each (12 items of each type were actually administered, so that the nine psychometrically best-performing items could be selected to form the final battery). Each item consisted of a target stimulus image depicting a 2D or 3D object (a “brick”), and four multiple-choice response images, one of which (the correct answer) showed the same object as the target, following an appropriate manipulation. Correct answers were summed to create subtest scores, from which composite scores were derived as described in Results. Participants completed the subtests in the following sequence. i) 2D Rotation: the 2D target object is rotated in the picture plane. ii) 2D Rotation/Visualisation combined: the rotating target is partially obscured behind an (immobile) occluding shape. iii) 2D Visualisation: the target remains static while the occluding shape changes location. iv) 3D Rotation/Visualisation combined: the object rotates freely in three dimensions. v) 3D Rotation: the 3D object rotates only in the picture plane. vi) 3D Visualisation: the target is a wireframe diagram, and the correct response is the “solid” object depicted. Examples of stimuli (targets and correct responses) are presented in Fig. 1, and these measures are described in greater detail in the Supplementary Methods online.

Two other cognitive measures were also available for this sample. The Mill Hill Vocabulary Scale^25^ was used as an index of verbal ability: across 33 trials, participants selected which of six multiple-choice options was closest in meaning to a target word. Non-verbal ability was assessed with Raven’s Progressive Matrices^26^, in which participants selected which of eight options completed a visual pattern, across 30 trials. Correct responses for each measure were summed and standardised, and the mean of these scores was used as a proxy of general cognitive ability (*g*). Participants completed these measures four years earlier than the Bricks battery, but since the genetic influences on *g* are highly stable over time^28^,^29^, this is unlikely to have influenced results. Where these measures were used as a control for domain-general cognitive processes, it should be noted that the verbal ability measure is probably an under-correction (as verbal ability is only a portion of *g*^22^), and that the non-verbal ability measure is in all likelihood an *over*-correction, as Raven’s Progressive Matrices have a substantial spatial component^30^.

Participants were contacted by post, but participated online via the TEDS websites. The measures administered at age 16 were implemented using the Flash browser plugin. The Bricks items were developed with “Building Bricks”, a web application developed for the purpose, and administered using the “psy.js” JavaScript library; both of these tools are open-source and freely available (see the Supplementary Methods online).

### Twin data

DZ twins share 50% of their segregating genes on average, while MZ twins share 100%, but environments are shared to approximately the same extent for both MZ and DZ twins. Genetic influence on a trait is therefore indicated by the degree to which the intrapair MZ correlation exceeds the DZ correlation, and cross-twin cross-trait correlations (i.e., the correlation between twin 1 on the first trait and twin 2 on the second) allow the genetic influences common to multiple traits to be estimated.

MZs and same-sex DZs are perfectly correlated for sex, and all twins are for age; it is therefore common practice to regress twin data on sex and age, to avoid the artificially inflated estimates of shared environmental influences which would otherwise result^31^. In addition, for each measure in the present study, outliers beyond 3 SD from the mean were removed, along with any data for those participants suspected to have suffered technical errors or to have responded randomly or carelessly (see the Supplementary Methods online). Participants with severe physical or psychological disabilities, or whose mothers had experienced serious perinatal complications, were also excluded from analysis. All variables were standardised, and since the Bricks variables were slightly skewed, a van der Waerden rank transformation^32^ was performed to ensure that all data were normally distributed, as required for the model-fitting procedures.

The study was approved by the appropriate King’s College London ethics committee, and was conducted in accordance with the approved guidelines. Participants provided informed consent.

### Model-fitting

The data were subjected to full-information maximum-likelihood (FIML) model-fitting procedures, accounting for missing data and combining both same- and opposite-sex DZ twins to maximise power. Univariate ACE models^33^ were fitted to the data, which use the expected genetic and environmental correlations between the twins (additive genetic influences correlating 1.0 for MZs and 0.5 for DZs; shared environment 1.0 for both; non-shared environment 0 for both) to apportion the variance into components attributable to: i) additive genetic influences (A); ii) shared (or “common”) environmental influences making people raised in the same family more similar to each other (C); and iii) non-shared (unique) environmental influences making them less similar (E, which also includes any measurement error). Individual components may be dropped in nested sub-models, but the full ACE models were used here despite C being non-significant for the Bricks measures, both because this tends to produce the most conservative heritability estimates, and for consistency with the other cognitive measures used (as C is significant for Raven’s Progressive Matrices; see Supplementary Table S15). All model-fitting was conducted using OpenMx^34^, an R package for structural equations.

Multivariate ACE model-fitting uses cross-twin cross-trait correlations^22^ to estimate the genetic and environmental sources of covariance, revealing the architecture underpinning two or more traits^35^. This calculates the genetic correlations (rA) between each pair of variables, which are independent from the heritability estimates of either trait, and indicate the degree to which they share genetic influences–i.e., common genes. A “correlated factors” solution then estimates common A, C and E influences, and thus allows phenotypic correlations to be decomposed into these sources of covariance (as in Fig. 2 and Supplementary Tables S16–S19). Alternatively, the (algebraically equivalent) Cholesky decomposition focuses instead on the *total* influences on each trait in sequence, and determines at each step the proportion of its A, C and E components that are shared with, or independent from, each variable. This process is analogous to stepwise multiple regression, accounting for the influences on each variable in turn, in order to determine the residual portions at each stage. Thus in bivariate models (as in Fig. 3 and Supplementary Tables S20–S23), path estimates show the proportion of each component that is common to both variables, and the proportion unique to the second variable. Similarly, trivariate and further extensions (as in Supplementary Figs S1 and S2 and Supplementary Tables S29–S36) indicate the influences in common to all variables, then those common to all but the first, and so on, and finally those influences unique to the last variable.

## Additional Information

**How to cite this article**: Shakeshaft, N. G. *et al.* Rotation is visualisation, 3D is 2D: using a novel measure to investigate the genetics of spatial ability. *Sci. Rep.* **6**, 30545; doi: 10.1038/srep30545 (2016).

## Supplementary Material

Supplementary Information

## Figures and Tables

**Table 1:** Twin correlations and approximated variance components.

	Intrapair twin correlations		Variance component estimates			Sample (numbers of pairs)	
	MZ	DZ	h^2^	c^2^	e^2^	MZ	DZ
Rotation	0.33 (0.26–0.41)	0.21 (0.14–0.28)	0.25	0.09	0.67	520	714
Rotation/Visualisation	0.38 (0.31–0.46)	0.22 (0.14–0.28)	0.34	0.05	0.62	521	714
Visualisation	0.45 (0.38–0.51)	0.22 (0.15–0.29)	0.45	0.00	0.55	516	711
2D	0.47 (0.40–0.53)	0.25 (0.18–0.31)	0.44	0.02	0.53	526	724
3D	0.41 (0.33–0.48)	0.20 (0.13–0.27)	0.41	0.00	0.59	508	697
Overall Bricks	0.56 (0.49–0.61)	0.27 (0.20–0.33)	0.56	0.00	0.44	522	720

Intraclass twin correlations (95% confidence intervals) for MZ and DZ twins, for the Bricks composites. Variance component estimates are heritability (h^2^: double the difference between the MZ and DZ correlations, constrained not to exceed the former–MZ twins are genetically identical, so heritability cannot exceed their correlation), shared environment (c^2^: the MZ correlation minus h^2^), and unique environment + error of measurement (e^2^: 1-h^2^-c^2^). Sample sizes shown are complete pairs, after exclusions and data cleaning.

**Table 2:** Univariate model-fitting results.

	A	C	E
Rotation	0.23 (0.03–0.40)	*0.10 (0.00*–*0.26)*	0.67 (0.60–0.75)
Rotation/Visualisation	0.34 (0.14–0.45)	*0.05 (0.00*–*0.20)*	0.62 (0.55–0.69)
Visualisation	0.43 (0.24–0.50)	*0.01 (0.00*–*0.16)*	0.56 (0.50–0.63)
2D	0.45 (0.27–0.52)	*0.02 (0.00*–*0.16)*	0.53 (0.48–0.60)
3D	0.41 (0.22–0.47)	*0.00 (0.00*–*0.15)*	0.59 (0.53–0.66)
Overall Bricks	0.55 (0.42–0.60)	*0.00 (0.00*–*0.11)*	0.45 (0.40–0.50)

Model-fitting estimates (95% confidence intervals) for additive genetic (A), shared environmental (C) and residual (E; i.e., non-shared environment and error) components of variance. Italicised estimates are non-significant (their confidence intervals include zero).
