# Supplementary material for: Rotation is visualisation, 3D is 2D: using a novel measure to investigate the genetics of spatial ability
Source: Sci Rep. 2016 Aug 1;6:30545. doi: 10.1038/srep30545 (PMC4967849; doi:10.1038/srep30545)
Supplement: Supplementary Information [file srep30545-s1.pdf]

Rotation is visualisation, 3D is 2D: using a novel measure to investigate the genetics of spatial ability

Nicholas G. Shakeshaft, Kaili Rimfield, Kerry L. Schofield, Saskia Selzam, Margherita Malanchini, Maja Rodic, Yulia Kovas & Robert Plomin

Supplementary Information

Supplementary methods – the Bricks battery

Rationale, development and description of the Bricks measures.

Figures

Fig. S1. Trivariate Cholesky decomposition path estimates: *g*, Rotation, Visualisation.

Fig. S2. Quadrivariate Cholesky decomposition path estimates: Verbal, non-verbal, 2D, 3D.

Tables

Table S1. Descriptive statistics.

Table S2. Internal consistency and test-retest reliability of Bricks measures.

Table S3. Subtest intercorrelations.

Table S4. Subtest factor analysis.

Table S5. Bricks correlations with other measures.

Table S6. Subtest intercorrelations, regressed on verbal ability.

Table S7. Subtest intercorrelations, regressed on non-verbal ability.

Table S8. Subtest intercorrelations, regressed on *g*.

Table S9. Functional composite intercorrelations, regressed on verbal ability.

Table S10. Functional composite intercorrelations, regressed on non-verbal ability.

Table S11. Functional composite intercorrelations, regressed on *g*.

Table S12. Dimensional composite correlation, regressed on other measures.

Table S13. Subtest factor analysis, regressed on other measures.

Table S14. Twin correlations and approximated variance components.

Table S15. Univariate model-fitting results.

Table S16. Decomposition of phenotypic correlations.

Table S17. Proportions of Bricks subtest correlations due to common genetic influences.

Table S18. Proportions of Bricks subtest correlations due to common non-shared environmental influences.

Table S19. Proportions of correlations with other measures due to common genetic influences.

Table S20. Bivariate Cholesky decomposition: Rotation, Visualisation.

Table S21. Bivariate Cholesky decomposition: Rotation, Rotation/Visualisation.

Table S22. Bivariate Cholesky decomposition: Visualisation, Rotation/Visualisation.

Table S23. Bivariate Cholesky decomposition: 2D, 3D.

Table S24. Correlations between influences on functional composites.

Table S25. Correlations between influences on dimensional composites.

Table S26. Genetic correlations among Bricks subtests.

Table S27. Non-shared environmental correlations among Bricks subtests.

Table S28. Genetic correlations with other measures.

Table S29. Trivariate Cholesky decomposition: verbal ability, Rotation, Visualisation.

Table S30. Trivariate Cholesky decomposition: non-verbal ability, Rotation, Visualisation.

Table S31. Trivariate Cholesky decomposition: *g*, Rotation, Visualisation.

Table S32. Trivariate Cholesky decomposition: verbal ability, 2D, 3D.

Table S33. Trivariate Cholesky decomposition: non-verbal ability, 2D, 3D.

Table S34. Trivariate Cholesky decomposition: *g*, 2D, 3D.

Table S35. Quadrivariate Cholesky decomposition: verbal, non-verbal, Rotation, Visualisation.

Table S36. Quadrivariate Cholesky decomposition: verbal, non-verbal, 2D, 3D.

Table S37. Fit statistics: univariate Bricks composite models.

Table S38. Fit statistics: bivariate Bricks composite models.

Table S39. Fit statistics: trivariate Bricks composite models.

Table S40. Fit statistics: quadrivariate Bricks composite models.

Supplementary methods – the Bricks battery

Rationale

As discussed in the main text, the literature on spatial abilities is inconsistent regarding the relationship between mental rotation and spatial visualisation, and between 2D and 3D stimuli. If rotation and visualisation were dissociable processes, it was reasoned that traditional 2D and 3D mental rotation stimuli may engage them differently. With 3D mental rotation stimuli, target objects commonly rotate freely in three dimensions, such that key identifiable features are out of view or disguised by foreshortening. However, with 2D stimuli, in which the object rotates only in the picture plane (i.e., as though rotating the whole image itself, rather than the object), full information about the object is always available, and there is no need to visualise missing or disguised features.

The Bricks battery was therefore developed to isolate rotation and visualisation cleanly, and to include stimuli depicting 2D objects with concealed features (as a closer match to common 3D stimuli), and 3D objects which do *not* obscure features (as with common 2D stimuli). In this way, the putative rotation and visualisation processes could be assessed both separately and together, equally in 2D and in 3D.

Design

Six subtests were conceived. Each consists of a series of items with a stimulus image containing a “target” object, and four multiple-choice response images, only one of which (the correct response) depicts the same object as the target, following a suitable transformation. Participants completed the subtests in the following order:

i) 2D Rotation: the most “natural” form of 2D rotation, in which the target (a two-dimensional object) is rotated only in the picture plane, and the target stimulus and correct response contain exactly the same information.

ii) 2D Rotation / Visualisation combined: to add the element of incomplete information commonly found in 3D stimuli, the target object is partially obscured behind an “occluder” - a square or circle quadrant partially obscuring the target. In the correct response, the target has rotated (in the picture plane) but the occluder is immobile.

iii) 2D Visualisation: the target remains entirely motionless and unchanged, but the occluder is in a different location in the correct response, thereby revealing a different portion of the target.

iv) 3D Rotation / Visualisation combined: the most “natural” form of 3D rotation, in which the target (a three-dimensional object, computer-generated and rendered with simple overhead “lighting”) has been rotated freely in three dimensions in the correct response.

v) 3D Rotation: corresponding to 2D rotation but with an image of an apparently three-dimensional object – in the correct response, the target is rotated only in the picture plane (i.e., as though the whole image had rotated, or the “camera” showing the scene had rotated on the spot). As with 2D rotation, the target stimulus and correct response therefore contain invariant information, with even the lighting and shadows remaining unchanged.

vi) 3D Visualisation: to assess visualisation without rotation, the target stimulus depicts a wireframe drawing of an object, and the correct response shows the “solid” version, otherwise unchanged. The participant must therefore use the available information to determine how the solid will appear (e.g., which features are in view from the current perspective and which are obscured by others).

Development

A JavaScript web application, “Building Bricks”, was developed to enable appropriate stimuli to be created for each subtest. This allows the creation of images of “bricks” (rectangular blocks, either 2D or 3D) of variable size, including one or more “studs” – protrusions of arbitrary length emerging from the main body of the brick, from the “top”, “bottom” or both. 2D bricks may be rotated in the picture plane, 3D bricks in any direction, and the camera may be rotated to simulate picture-plane rotation for 3D objects. Occluders

(squares or circle quadrants) of arbitrary size may be added to any corner of the image. Various other options such as colours or camera distances may be altered as required, and bricks may be presented in wireframe or solid form.

This software is freely available online under the open-source MIT license, and researchers are welcome to experiment with it to see how the constructs were operationalised, or to create their own items. It is accessible via this page: https://www.forepsyte.com/resources/public

For each subtest, 12 items of varying difficulty were created and administered, but with a view to reducing this to 9 items post hoc before the calculation of scores. This allowed the final selection to be approximately equated for difficulty between subtests, and for 'experimental' items (e.g., those with potentially counterintuitive responses) to be included in the initial battery before being discarded on the basis of their psychometric properties. Examples of stimulus images and the corresponding correct responses are shown in Fig. 1.

Participants completed the Bricks battery online, via a website created for the purpose using the open-source “psy.js” JavaScript library, which was developed specifically for the administration of psychometric measures such as questionnaires and cognitive tests. This library is also freely available at the link above.

Procedure

For each subtest, participants read appropriate instructions, completed two simple practice items (which provided feedback and clarification of the subtest rules), and then completed the test items in a fixed sequence of approximately increasing difficulty (selected based on pilot work). A time limit of 20 seconds was allowed for each item – the time remaining was displayed to participants via a timer at the top right of the screen. If participants made four consecutive incorrect responses, they were discontinued from the current subtest and began the next. Including the time spent reading instructions and reviewing practice items, the battery typically took 20-25 minutes to complete.

Data cleaning and scoring

After the participant exclusions described in the main text (e.g., excluding those with relevant severe disabilities), and prior to the data preparation procedures described (outlier removal, etc.), additional exclusions were made on the basis of suspected random or thoughtless responding. Conservative cut-offs were used to identify participants with very low variability in their responses – 3SD below the mean, indicating that they had clicked on the same response option repeatedly for most or all items – or with mean reaction times of less than one second per item. Participants falling below these cut-offs were excluded from analysis.

For each item, a score of 1 was awarded for a correct response, or 0 for incorrect responses, no response or the item being skipped due to discontinuation. Scores from the nine items in the final battery were summed to yield subtest scores. These individual subtest scores were then cleaned and combined into “functional”, “dimensional” and “overall Bricks” composites, as described in the main text.

Fig. S1. Trivariate Cholesky decomposition path estimates: *g*, Rotation, Visualisation.

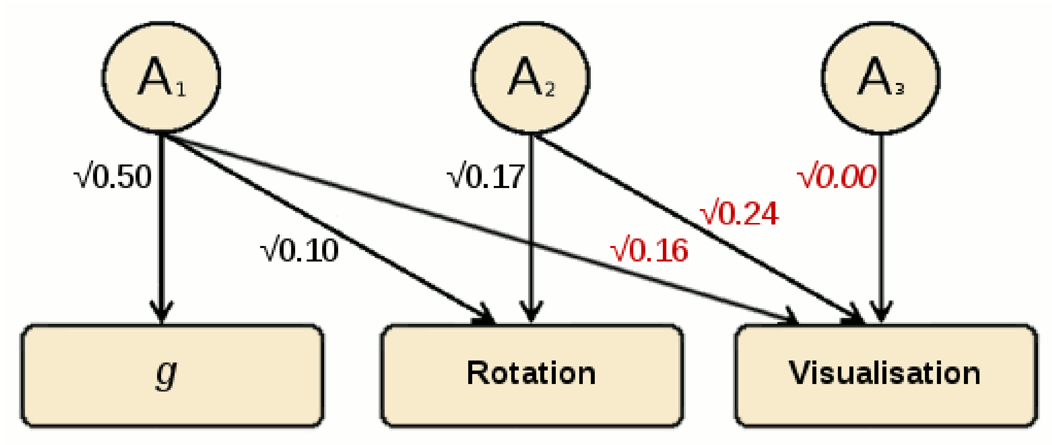

Path estimates (standardised) for the structure of additive genetic influences on *g*, Rotation and Visualisation (see Table S31 for more details). The paths in red indicate the genetic influences on Visualisation (the last variable in the model): i) those common to all three variables; ii) those shared only between Rotation and Visualisation but not with *g* (suggesting influences specific to spatial ability); and iii) those unique to Visualisation alone. The latter (italicised) is non-significant – i.e., all genetic influences on Visualisation are shared with Rotation.

Fig. S2. Quadrivariate Cholesky decomposition path estimates: Verbal, non-verbal, 2D, 3D.

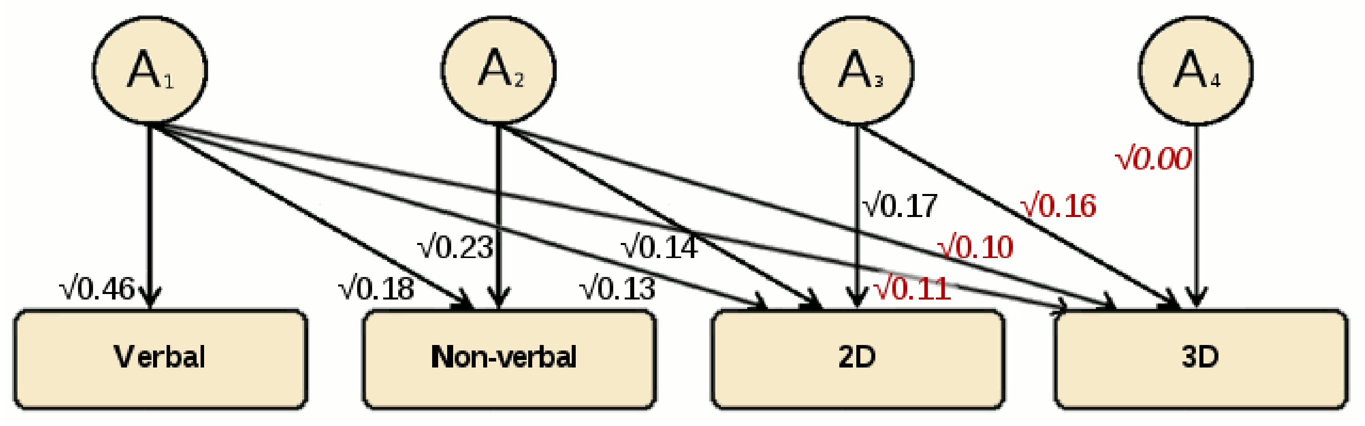

Path estimates (standardised) for the structure of additive genetic influences on verbal ability, non-verbal ability, and the 2D and 3D Bricks composites (see Table S36 for more details). The paths in red indicate the genetic influences on 3D (the last variable in the model): i) those common to all four variables; ii) those shared between non-verbal ability, and the 2D and 3D Bricks composites, but not with verbal ability; iii) those shared only between 2D and 3D but not with verbal or non-verbal ability (suggesting influences specific to spatial ability); and iv) those unique to 3D alone. The latter (italicised) is non-significant – i.e., all genetic influences on 3D are shared with 2D.

Table S1. Descriptive statistics.

	N	Whole sample	Males	Females	MZs	DZs	Sex	Zyg	Sex x zyg	R^2
2D Rotation	1451	5.58 (1.68)	5.77 (1.75)	5.47 (1.64)	5.47. (1.72)	5.65 (1.65)	12.73 **	2.80	1.19	0.01
2D Rotation / Visualisation	1443	4.87 (1.94)	5.16 (2.01)	4.70 (1.89)	4.76 (1.96)	4.95 (1.93)	19.48 **	2.35	1.92	0.02
2D Visualisation	1434	5.05 (2.02)	5.32 (1.99)	4.90 (2.02)	5.08 (2.00)	5.04 (2.03)	15.92 **	0.37	0.24	0.01
3D Rotation	1403	6.41 (1.69)	6.53 (1.71)	6.34 (1.67)	6.44 (1.67)	6.39 (1.70)	4.91 *	0.56	0.16	0.00
3D Rotation / Visualisation	1426	5.58 (1.34)	5.85 (1.42)	5.42 (1.26)	5.56 (1.33)	5.59 (1.34)	33.70 **	0.00	1.88	0.02
3D Visualisation	1427	5.61 (2.07)	6.01 (2.01)	5.37 (2.07)	5.56 (2.09)	5.64 (2.06)	36.40 **	0.14	1.48	0.03
Rotation	1435	5.99 (1.34)	6.17 (1.36)	5.88 (1.32)	5.97 (1.36)	6.00 (1.33)	17.40 **	0.02	1.92	0.01
Rotation / Visualisation	1440	5.23 (1.36)	5.52 (1.43)	5.06 (1.29)	5.17 (1.34)	5.27 (1.37)	39.00 **	0.78	2.63	0.03
Visualisation	1429	5.35 (1.69)	5.69 (1.61)	5.15 (1.70)	5.35 (1.65)	5.35 (1.71)	35.83 **	0.07	0.00	0.03
2D	1451	5.17 (1.42)	5.44 (1.43)	5.02 (1.39)	5.12 (1.41)	5.21 (1.43)	31.41 **	0.70	1.04	0.02
3D	1414	5.88 (1.28)	6.15 (1.30)	5.72 (1.25)	5.89 (1.24)	5.87 (1.31)	45.88 **	0.31	1.81	0.03
Overall Bricks	1443	5.52 (1.21)	5.79 (1.23)	5.37 (1.18)	5.49 (1.19)	5.54 (1.23)	46.90 **	0.08	1.28	0.03
Verbal	1442	15.61 (4.00)	15.72 (4.05)	15.55 (3.97)	15.30 (3.98)	15.82 (4.00)	0.01	3.40	0.02	0.00
Non-verbal	1437	14.06 (3.63)	14.33 (3.76)	13.91 (3.54)	13.95 (3.61)	14.14 (3.64)	3.40	0.01	0.82	0.00
g	1439	0.06 (0.97)	0.12 (0.99)	0.03 (0.96)	0.00 (0.97)	0.10 (0.98)	1.26	2.12	0.84	0.00

Mean scores (standard deviations) for the whole sample, separately by sex, and for MZ and DZ twins, for the six Bricks subtests, the three functional and two dimensional composites, the single overall Bricks mean, and the other cognitive measures. N = sample size (the sample shown is fully independent, selecting one individual randomly per twin pair). ANOVA performed on cleaned, normality-transformed data to test effects of sex and zygosity. Results = F statistic; ** = $p < 0.01$; * = $p < 0.05$; R^2 = proportion of variance explained by sex, zygosity and their interaction.

Table S2. Internal consistency and test-retest reliability of Bricks measures.

	**Consistency**		**Test-retest reliability**
	Alpha	N	Pearson's *r* (N = 45, p < 0.01)
2D Rotation	0.45	1453	0.42
2D Rotation / Visualisation	0.60	1443	0.63
2D Visualisation	0.63	1434	0.52
3D Rotation	0.62	1429	0.59
3D Rotation / Visualisation	0.47	1431	0.51
3D Visualisation	0.71	1427	0.57
Rotation	0.63	1429	0.62
Rotation / Visualisation	0.66	1431	0.75
Visualisation	0.75	1427	0.62
2D	0.74	1434	0.77
3D	0.78	1427	0.67
Overall Bricks	0.85	1427	0.83

Consistency (Cronbach's alpha) and test-retest reliability (Pearson's *r*) for the six Bricks subtests, the three functional and two dimensional composites, and the single overall mean. The consistency sample is fully independent, with one individual selected randomly from each twin pair. Test-retest reliability was assessed with a separate pilot sample.

Table S3. Subtest intercorrelations.

		**Overall Bricks**	2D R	2D R / V	2D V	3D R	3D R / V	3D V
2D Rotation	*r*	**0.57**	1					
	N	***1441***	*1451*					
2D Rotation / Visualisation	*r*	**0.71**	0.31	1				
	N	***1433***	*1441*	*1443*				
2D Visualisation	*r*	**0.69**	0.27	0.42	1			
	N	***1424***	*1432*	*1434*	*1434*			
3D Rotation	*r*	**0.62**	0.25	0.34	0.35	1		
	N	***1401***	*1401*	*1403*	*1403*	*1403*		
3D Rotation / Visualisation	*r*	**0.59**	0.27	0.32	0.31	0.26	1	
	N	***1420***	*1424*	*1426*	*1426*	*1403*	*1426*	
3D Visualisation	*r*	**0.68**	0.27	0.37	0.34	0.34	0.38	1
	N	***1417***	*1425*	*1427*	*1427*	*1401*	*1422*	*1427*

Correlations (Pearson's *r*) between the six subtests, and between each subtest and the overall Bricks mean. The sample is fully independent, with one individual selected randomly from each twin pair. R = Rotation; R / V = Rotation and Visualisation; V = Visualisation. All correlations significant at p < 0.0001.

Table S4. Subtest factor analysis.

	Factor loading
2D Rotation	0.57
2D Rotation / Visualisation	0.70
2D Visualisation	0.68
3D Rotation	0.65
3D Rotation / Visualisation	0.63
3D Visualisation	0.68

Factor loadings of Bricks subtests on the first (and only) principal component produced by factor analysis of the six subtest scores. The factor accounts for 2.56 eigenvalues, 42.6% of total variance.

Table S5. Bricks correlations with other measures.

		Mill Hill (verbal)	Raven's Matrices (non-verbal)	*g*
Rotation	*r*	0.13	0.35	0.30
	N	*1414*	*1410*	*1412*
Rotation / Visualisation	*r*	0.24	0.45	0.41
	N	*1419*	*1414*	*1416*
Visualisation	*r*	0.21	0.44	0.39
	N	*1410*	*1405*	*1408*
2D	*r*	0.19	0.44	0.39
	N	*1430*	*1425*	*1427*
3D	*r*	0.22	0.46	0.41
	N	*1393*	*1389*	*1391*
Overall Bricks	*r*	0.22	0.50	0.44
	N	*1422*	*1417*	*1419*

Correlations (Pearson's *r*) with other cognitive measures for the three functional and two dimensional Bricks composites, and the single overall mean. Mill Hill and Raven's Matrices correlate $r = 0.31$ with each other in this sample (N = 1420). All correlations significant at $p < 0.0001$.

N.B. The Rotation correlations with each other measure are significantly lower than those of Visualisation (all $p < 0.01$); however, since the 'Rotation / Visualisation combined' correlations do *not* differ significantly from those of Visualisation (despite the 'Rotation / Visualisation combined' conditions including both elements), this seems most likely to be related to the slightly lower reliability of one of the Rotation subtests (2D rotation) compared to the others, coupled with the highly-powered sample size, rather than representing a theoretically meaningful difference.

Table S6. Subtest intercorrelations, regressed on verbal ability.

		Overall Bricks	2D R	2D R / V	2D V	3D R	3D R / V	3D V
2D Rotation	*r*	**0.57**	1					
	N	***1420***	*1430*					
2D Rotation / Visualisation	*r*	**0.70**	0.29	1				
	N	***1412***	*1420*	*1422*				
2D Visualisation	*r*	**0.68**	0.26	0.39	1			
	N	***1403***	*1411*	*1413*	*1413*			
3D Rotation	*r*	**0.62**	0.24	0.33	0.33	1		
	N	***1380***	*1380*	*1382*	*1382*	*1382*		
3D Rotation / Visualisation	*r*	**0.57**	0.26	0.29	0.29	0.25	1	
	N	***1399***	*1403*	*1405*	*1405*	*1382*	*1405*	
3D Visualisation	*r*	**0.66**	0.26	0.34	0.32	0.32	0.36	1
	N	***1396***	*1404*	*1406*	*1406*	*1380*	*1401*	*1406*

Correlations (Pearson's *r*) between the six subtest residuals after regression on verbal ability (Mill Hill scores), and between each subtest and the overall Bricks mean. R = Rotation; R / V = Rotation and Visualisation; V = Visualisation. All correlations significant at $p < 0.0001$.

Table S7. Subtest intercorrelations, regressed on non-verbal ability.

		Overall Bricks	2D R	2D R / V	2D V	3D R	3D R / V	3D V
2D Rotation	*r*	**0.52**	1					
	N	***1415***	*1425*					
2D Rotation / Visualisation	*r*	**0.65**	0.22	1				
	N	***1407***	*1415*	*1417*				
2D Visualisation	*r*	**0.64**	0.19	0.33	1			
	N	***1398***	*1406*	*1408*	*1408*			
3D Rotation	*r*	**0.58**	0.18	0.25	0.27	1		
	N	***1376***	*1376*	*1378*	*1378*	*1378*		
3D Rotation / Visualisation	*r*	**0.50**	0.19	0.21	0.20	0.17	1	
	N	***1394***	*1398*	*1400*	*1400*	*1378*	*1400*	
3D Visualisation	*r*	**0.60**	0.18	0.25	0.24	0.25	0.28	1
	N	***1391***	*1399*	*1401*	*1401*	*1376*	*1396*	*1401*

Correlations (Pearson's *r*) between the six subtest residuals after regression on non-verbal ability (Raven's Matrices scores), and between each subtest and the overall Bricks mean. R = Rotation; R / V = Rotation and Visualisation; V = Visualisation. All correlations significant at $p < 0.0001$.

Table S8. Subtest intercorrelations, regressed on *g*.

		**Overall Bricks**	2D R	2D R / V	2D V	3D R	3D R / V	3D V
2D Rotation	*r*	**0.55**	1					
	N	***1417***	*1427*					
2D Rotation / Visualisation	*r*	**0.66**	0.24	1				
	N	***1409***	*1417*	*1419*				
2D Visualisation	*r*	**0.65**	0.21	0.35	1			
	N	***1400***	*1408*	*1410*	*1410*			
3D Rotation	*r*	**0.59**	0.21	0.28	0.29	1		
	N	***1378***	*1378*	*1380*	*1380*	*1380*		
3D Rotation / Visualisation	*r*	**0.52**	0.21	0.23	0.23	0.19	1	
	N	***1396***	*1400*	*1402*	*1402*	*1380*	*1402*	
3D Visualisation	*r*	**0.62**	0.20	0.27	0.26	0.27	0.30	1
	N	***1393***	*1401*	*1403*	*1403*	*1378*	*1398*	*1403*

Correlations (Pearson's *r*) between the six subtest residuals after regression on *g* (the mean of verbal and non-verbal ability scores), and between each subtest and the overall Bricks mean. R = Rotation; R / V = Rotation and Visualisation; V = Visualisation. All correlations significant at p < 0.0001.

Table S9. Functional composite intercorrelations, regressed on verbal ability.

		Rotation	Rotation / Visualisation	Visualisation
Rotation	*r*	1		
	N	*1414*		
Rotation / Visualisation	*r*	0.44	1	
	N	*1402*	*1419*	
Visualisation	*r*	0.44	0.51	1
	N	*1392*	*1407*	*1410*

Correlations (Pearson's *r*) between the three functional Bricks composite residuals after regression on verbal ability (Mill Hill scores). All correlations significant at p < 0.0001.

Table S10. Functional composite intercorrelations, regressed on non-verbal ability.

		Rotation	Rotation / Visualisation	Visualisation
Rotation	*r*	1		
	N	*1410*		
Rotation / Visualisation	*r*	0.35	1	
	N	*1398*	*1414*	
Visualisation	*r*	0.36	0.42	1
	N	*1388*	*1402*	*1405*

Correlations (Pearson's *r*) between the three functional Bricks composite residuals after regression on non-verbal ability (Raven's Matrices scores). All correlations significant at p < 0.0001.

Table S11. Functional composite intercorrelations, regressed on *g*.

		Rotation	Rotation / Visualisation	Visualisation
Rotation	*r*	1		
	N	*1412*		
Rotation / Visualisation	*r*	0.38	1	
	N	*1400*	*1416*	
Visualisation	*r*	0.38	0.44	1
	N	*1391*	*1405*	*1408*

Correlations (Pearson's *r*) between the three functional Bricks composite residuals after regression on *g* (the mean of verbal and non-verbal ability scores). All correlations significant at $p < 0.0001$.

Table S12. Dimensional composite correlation, regressed on other measures.

		**Regressed variable**		
		Mill Hill (verbal)	Raven's Matrices (non-verbal)	*g*
2D and 3D	*r*	0.54	0.44	0.47
	N	*1392*	*1388*	*1390*

Correlation between 2D and 3D dimensional Bricks composites, after regression on verbal ability, non-verbal ability or *g* (their mean). All correlations significant at $p < 0.0001$.

Table S13. Subtest factor analysis, regressed on other measures.

	**Regressed variable**		
	Mill Hill (verbal)	Raven's Matrices (non-verbal)	*g*
2D Rotation	0.57	0.51	0.54
2D Rotation / Visualisation	0.69	0.64	0.65
2D Visualisation	0.67	0.62	0.64
3D Rotation	0.64	0.61	0.62
3D Rotation / Visualisation	0.62	0.54	0.57
3D Visualisation	0.67	0.62	0.63
Variance explained	*41.3% (2.48 eigenvalues)*	*35.1% (2.10 eigenvalues)*	*37.0% (2.22 eigenvalues)*

Factor loadings of Bricks subtests on the first (and only) principal component produced by factor analysis of the six subtest scores, after regression on verbal ability, non-verbal ability or *g*.

Table S14. Twin correlations and approximated variance components.

	**Intrapair twin correlations**		**Variance component estimates**			**Sample (numbers of pairs)**	
	MZ	DZ	h^2	c^2	e^2	MZ	DZ
2D Rotation	0.21 (0.12 – 0.29)	0.11 (0.03 – 0.18)	0.20	0.01	0.79	528	722
2D Rotation / Visualisation	0.28 (0.20 – 0.36)	0.16 (0.09 – 0.23)	0.24	0.04	0.72	525	718
2D Visualisation	0.24 (0.16 – 0.32)	0.17 (0.10 – 0.24)	0.14	0.10	0.76	521	713
3D Rotation	0.21 (0.13 – 0.29)	0.13 (0.06 – 0.21)	0.16	0.05	0.79	502	684
3D Rotation / Visualisation	0.27 (0.19 – 0.35)	0.12 (0.05 – 0.20)	0.27	0.00	0.73	516	704
3D Visualisation	0.34 (0.26 – 0.41)	0.08 (0.01 – 0.15)	0.34	0.00	0.66	517	710
Verbal	0.48 (0.43 – 0.54)	0.27 (0.21 – 0.32)	0.43	0.05	0.52	729	1173
Non-verbal	0.51 (0.45 – 0.56)	0.32 (0.26 – 0.37)	0.39	0.12	0.49	700	1086
g	0.58 (0.52 – 0.62)	0.34 (0.29 – 0.39)	0.47	0.10	0.42	697	1084

Intraclass twin correlations (95% confidence intervals) for MZ and DZ twins, for the six Bricks subtests and for verbal ability (Mill Hill), non-verbal ability (Raven's Matrices) and *g* (their mean). For Bricks composites, see Table 1. Variance component estimates are heritability (h^2: double the difference between the MZ and DZ correlations, constrained not to exceed the former – MZ twins are genetically identical, so heritability cannot exceed their correlation), shared environment (c^2: the MZ correlation minus h^2), and unique environment + error of measurement (e^2: 1 - h^2 - c^2). Sample sizes shown are complete pairs, after exclusions and data cleaning.

Table S15. Univariate model-fitting results.

	A	C	E
2D Rotation	*0.19 (0.00 – 0.28)*	*0.01 (0.00 – 0.17)*	0.80 (0.72 – 0.88)
2D Rotation / Visualisation	0.23 (0.03 – 0.34)	*0.04 (0.00 – 0.20)*	0.73 (0.66 – 0.81)
2D Visualisation	*0.13 (0.00 – 0.31)*	*0.11 (0.00 – 0.24)*	0.76 (0.69 – 0.84)
3D Rotation	*0.15 (0.00 – 0.29)*	*0.06 (0.00 – 0.21)*	0.79 (0.71 – 0.87)
3D Rotation / Visualisation	0.26 (0.07 – 0.33)	*0.00 (0.00 – 0.14)*	0.74 (0.67 – 0.81)
3D Visualisation	0.30 (0.20 – 0.36)	*0.00 (0.00 – 0.07)*	0.70 (0.64 – 0.77)
Verbal	0.46 (0.32 – 0.55)	*0.04 (0.00 – 0.15)*	0.50 (0.45 – 0.55)
Non-verbal	0.40 (0.28 – 0.54)	0.12 (0.01 – 0.23)	0.48 (0.43 – 0.53)
g	0.49 (0.36 – 0.62)	*0.10 (0.00 – 0.21)*	0.41 (0.37 – 0.46)

Model-fitting estimates (95% confidence intervals) for additive genetic (A), shared environmental (C) and residual (E; i.e., non-shared environment and error) components of variance, for the six Bricks subtests and for verbal ability (Mill Hill), non-verbal ability (Raven's Matrices) and *g* (their mean). For Bricks composites, see Table 2. Italicised estimates are non-significant (their confidence intervals include zero).

Table S16. Decomposition of phenotypic correlations.

Variables in model	Variance component estimates		
	A	C	E
Rotation<br>Rotation / Visualisation	0.80<br>(0.54 – 0.89)	*-0.02*<br>*(-0.06 – 0.19)*	0.22<br>(0.13 – 0.32)
Rotation<br>Visualisation	0.71<br>(0.43 – 0.85)	*-0.00*<br>*(-0.09 – 0.22)*	0.29<br>(0.20 – 0.39)
Visualisation<br>Rotation / Visualisation	0.74<br>(0.47 – 0.85)	*0.01*<br>*(-0.06 – 0.22)*	0.26<br>(0.18 – 0.35)
2D<br>3D	0.79<br>(0.60 – 0.86)	*-0.01*<br>*(-0.04 – 0.15)*	0.22<br>(0.15 – 0.30)

Bivariate correlated factors solutions of four models: three between the functional Bricks composites, and one between the dimensional composites. Results indicate the phenotypic correlations between the two composites in each model, decomposed into proportions attributable to additive genetic (A), shared environmental (C) or non-shared environmental/error (E) components (with 95% confidence intervals). The proportions explained reflect the correlation between the traits for that component, weighted by the two univariate component estimates – for example, the proportion of the phenotypic correlation due to A equals the genetic correlation weighted by the product of the square roots of the two univariate heritabilities estimated by the model. Italicised estimates are non-significant (their CIs include zero). Totals may exceed 1.00 due to rounding.

Table S17. Proportions of Bricks subtest correlations due to common genetic influences.

	2D R	2D R / V	2D V	3D R	3D R / V	3D V
2D Rotation	1					
2D Rotation / Visualisation	0.82<br>(0.48 – 0.97)	1				
2D Visualisation	0.73<br>(0.28 – 1.01)	0.47<br>(0.12 – 0.80)	1			
3D Rotation	0.60<br>(0.02 – 1.05)	0.69<br>(0.29 – 0.95)	*0.21*<br>*(-0.13 – 0.66)*	1		
3D Rotation / Visualisation	0.91<br>(0.50 – 1.09)	0.79<br>(0.36 – 1.04)	0.75<br>(0.37 – 0.94)	*0.50*<br>*(-0.02 – 0.89)*	1	
3D Visualisation	0.86<br>(0.52 – 1.09)	0.76<br>(0.50 – 0.90)	0.79<br>(0.46 – 0.96)	0.62<br>(0.29 – 0.82)	0.65<br>(0.30 – 0.82)	1

Bivariate correlated factors solutions, indicating the proportions of the phenotypic correlations between subtests due to common genetic influences (with 95% confidence intervals). Italicised estimates are non-significant (their CIs include zero). R = Rotation; R / V = Rotation and Visualisation; V = Visualisation.

N.B. The figures shown are proportions of the total covariance, so the two lower and non-significant estimates in this table reflect the correspondingly higher non-shared environment components (Table S17) for those associations (and the wide CIs), rather than a meaningful distinction from the other correlations.

Table S18. Proportions of Bricks subtest correlations due to common non-shared environmental influences.

	2D R	2D R / V	2D V	3D R	3D R / V	3D V
2D Rotation	1					
2D Rotation / Visualisation	0.18<br>(0.03 – 0.34)	1				
2D Visualisation	*0.18*<br>*(-0.00 – 0.36)*	0.31<br>(0.18 – 0.45)	1			
3D Rotation	*0.23*<br>*(-0.02 – 0.47)*	0.20<br>(0.05 – 0.36)	0.45<br>(0.28 – 0.63)	1		
3D Rotation / Visualisation	*0.10*<br>*(-0.09 – 0.29)*	*0.14*<br>*(-0.03 – 0.33)*	0.28<br>(0.12 – 0.45)	0.44<br>(0.23 – 0.66)	1	
3D Visualisation	*0.16*<br>*(-0.04 – 0.37)*	0.23<br>(0.10 – 0.36)	0.17<br>(0.04 – 0.30)	0.34<br>(0.19 – 0.49)	0.32<br>(0.18 – 0.48)	1

Bivariate correlated factors solutions, indicating the proportions of the phenotypic correlations between subtests due to common non-shared environmental influences (with 95% confidence intervals). Italicised estimates are non-significant (their CIs include zero). R = Rotation; R / V = Rotation and Visualisation; V = Visualisation.

Table S19. Proportions of correlations with other measures due to common genetic influences.

	Mill Hill (verbal)	Raven's Matrices (non-verbal)	*g*
Rotation	1.24<br>(0.50 – 1.90	0.56<br>(0.26 – 0.89)	0.69<br>(0.34 – 1.05)
Rotation / Visualisation	0.79<br>(0.35 – 1.23)	0.75<br>(0.50 – 1.00)	0.77<br>(0.51 – 1.03)
Visualisation	1.00<br>(0.49 – 1.40)	0.58<br>(0.33 – 0.82)	0.66<br>(0.40 – 0.92)
2D	1.08<br>(0.56 – 1.47)	0.74<br>(0.50 – 0.98)	0.80<br>(0.54 – 1.05)
3D	0.97<br>(0.54 – 1.32)	0.59<br>(0.36 – 0.81)	0.69<br>(0.45 – 0.92)
Overall Bricks	0.99<br>(0.64 – 1.29)	0.72<br>(0.52 – 0.90)	0.77<br>(0.57 – 0.95)

Bivariate correlated factors solutions, indicating the proportions of the phenotypic correlations (with 95% confidence intervals) between each Bricks composite and other cognitive measures which are attributable to common genetic influences.

N.B. the proportions above unity (with verbal ability) are offset by negative environmental contributions, but the wide CIs preclude any meaningful interpretations.

Table S20. Bivariate Cholesky decomposition: Rotation, Visualisation.

	**Genetic paths**		**Shared environment paths**		**Non-shared environment paths**	
	Rotation	Visualisation	Rotation	Visualisation	Rotation	Visualisation
1. Rotation	0.25 (0.11 – 0.38)		*0.09 (0.00 – 0.21)*		0.65 (0.57 – 0.73)	
2. Visualisation	0.44 (0.20 – 0.50)	*0.00 (0.00 – 0.18)*	*0.00 (0.00 – 0.09)*	*0.00 (0.00 – 0.11)*	0.03 (0.01 – 0.05)	0.53 (0.46 – 0.60)

Path estimates (standardised and squared, with 95% confidence intervals) for bivariate ACE Cholesky decomposition. The influences on the first entered variable (Rotation) are as in the univariate model for that variable (precise estimates vary between models), but those on the second (Visualisation) are decomposed into influences shared with the first variable, and those unique to the second. Italicised estimates are non-significant (their CIs include zero).

Table S21. Bivariate Cholesky decomposition: Rotation, Rotation/Visualisation.

	**Genetic paths**		**Shared environment paths**		**Non-shared environment paths**	
	Rotation	Rotation / Visualisation	Rotation	Rotation / Visualisation	Rotation	Rotation / Visualisation
1. Rotation	0.34 (0.19 – 0.42)		*0.03 (0.00 – 0.15)*		0.62 (0.55 – 0.70)	
2. Rotation / Visualisation	0.41 (0.25 – 0.47)	*0.00 (0.00 – 0.09)*	*0.00 (0.00 – 0.12)*	*0.00 (0.00 – 0.09)*	0.02 (0.01 – 0.03)	0.57 (0.50 – 0.64)

Path estimates (standardised and squared, with 95% confidence intervals) for bivariate ACE Cholesky decomposition. The influences on the first entered variable (Rotation) are as in the univariate model for that variable (precise estimates vary between models), but those on the second (Rotation / Visualisation combined) are decomposed into influences shared with the first variable, and those unique to the second. Italicised estimates are non-significant (their CIs include zero).

Table S22. Bivariate Cholesky decomposition: Visualisation, Rotation/Visualisation.

	**Genetic paths**		**Shared environment paths**		**Non-shared environment paths**	
	Visualisation	Rotation / Visualisation	Visualisation	Rotation / Visualisation	Visualisation	Rotation / Visualisation
1. Visualisation	0.44 (0.26 – 0.49)		*0.00 (0.00 – 0.14)*		0.56 (0.49 – 0.63)	
2. Rotation / Visualisation	0.32 (0.17 – 0.45)	*0.02 (0.00 – 0.13)*	*0.03 (0.00 – 0.18)*	*0.01 (0.00 – 0.10)*	0.03 (0.01 – 0.06)	0.58 (0.51 – 0.65)

Path estimates (standardised and squared, with 95% confidence intervals) for bivariate ACE Cholesky decomposition. The influences on the first entered variable (Visualisation) are as in the univariate model for that variable (precise estimates vary between models), but those on the second (Rotation / Visualisation combined) are decomposed into influences shared with the first variable, and those unique to the second. Italicised estimates are non-significant (their CIs include zero).

Table S23. Bivariate Cholesky decomposition: 2D, 3D.

	**Genetic paths**		**Shared environment paths**		**Non-shared environment paths**	
	2D	3D	2D	3D	2D	3D
1. 2D	0.45 (0.31 – 0.52)		*0.02 (0.00 – 0.14)*		0.53 (0.46 – 0.60)	
2. 3D	0.42 (0.28 – 0.48)	*0.00 (0.00 – 0.08)*	*0.00 (0.00 – 0.12)*	*0.00 (0.00 – 0.07)*	0.03 (0.01 – 0.05)	0.54 (0.47 – 0.61)

Path estimates (standardised and squared, with 95% confidence intervals) for bivariate ACE Cholesky decomposition. The influences on the first entered variable (2D) are as in the univariate model for that variable (precise estimates vary between models), but those on the second (3D) are decomposed into influences shared with the first variable, and those unique to the second. Italicised estimates are non-significant (their CIs include zero).

Table S24. Correlations between influences on functional composites.

	**Genetic correlations (rA)**		**Shared environment correlations (rC)**		**Non-shared environment correlations (rE)**	
	Rotation	Rotation / Visualisation	Rotation	Rotation / Visualisation	Rotation	Rotation / Visualisation
Rotation / Visualisation	1.00 (0.88 – 1.00)		*-1.00 (-1.00 – 1.00)*		0.17 (0.11 – 0.24)	
Visualisation	1.00 (0.74 – 1.00)	0.97 (0.95 – 1.00)	*-1.00 (-1.00 – 1.00)*	*0.86 (-1.00 – 1.00)*	0.23 (0.16 – 0.30)	0.23 (0.16 – 0.30)

Genetic, shared and non-shared environmental correlations (95% confidence intervals) between the functional Bricks composites. Italicised estimates are non-significant.

Table S25. Correlations between influences on dimensional composites.

	**Genetic correlation (rA)**	**Shared environment correlation (rC)**	**Non-shared environment correlation (rE)**
2D and 3D	1.00 (0.90 – 1.00)	*-1.00 (-1.00 – 1.00)*	0.22 (0.16 – 0.29)

Genetic, shared and non-shared environmental correlations (95% confidence intervals) between the dimensional Bricks composites. Italicised estimates are non-significant.

Table S26. Genetic correlations among Bricks subtests.

	2D R	2D R / V	2D V	3D R	3D R / V	3D V
2D Rotation	1					
2D Rotation / Visualisation	1.00 (0.83 – 1.00)	1				
2D Visualisation	1.00 (0.74 – 1.00)	1.00 (0.75 – 1.00)	1			
3D Rotation	0.86 (0.04 – 1.00)	1.00 (0.79 – 1.00)	0.61 (-0.97 – 1.00)	1		
3D Rotation / Visualisation	1.00 (0.77 – 1.00)	0.95 (0.94 – 1.00)	1.00 (0.69 – 1.00)	0.63 (-0.09 – 1.00)	1	
3D Visualisation	0.96 (0.95 – 1.00)	1.00 (1.00 – 1.00)	1.00 (0.91 – 1.00)	0.95 (0.76 – 1.00)	0.86 (0.65 – 1.00)	1

Genetic correlations (95% confidence intervals) among the individual Bricks subtests. R = Rotation; R / V = Rotation and Visualisation; V = Visualisation.

N.B. Two of these (3D Rotation's correlations with 2D Visualisation and with 3D Rotation/Visualisation) are technically non-significant, with CIs including zero; but given the high point estimates, and since this subtest's genetic correlations with other subtests have generally wider CIs than others, it seems likely that this reflects differences in the reliability of the subtests (or indeed chance differences) rather than a meaningful distinction from the other associations.

Table S27. Non-shared environmental correlations among Bricks subtests.

	2D R	2D R / V	2D V	3D R	3D R / V	3D V
2D Rotation	1					
2D Rotation / Visualisation	0.07 (0.01 – 0.14)	1				
2D Visualisation	*0.06 (-0.00 – 0.13)*	0.15 (0.09 – 0.22)	1			
3D Rotation	*0.07 (-0.01 – 0.14)*	0.09 (0.02 – 0.16)	0.19 (0.12 – 0.26)	1		
3D Rotation / Visualisation	*0.03 (-0.03 – 0.10)*	*0.06 (-0.01 – 0.13)*	0.11 (0.05 – 0.18)	0.15 (0.08 – 0.23)	1	
3D Visualisation	*0.06 (-0.01 – 0.13)*	0.12 (0.05 – 0.19)	0.08 (0.02 – 0.15)	0.16 (0.09 – 0.23)	0.16 (0.09 – 0.23)	1

Genetic correlations (95% confidence intervals) among the individual Bricks subtests. R = Rotation; R / V = Rotation and Visualisation; V = Visualisation.

N.B. Most subtests have modest non-shared environmental influences in common. Some of these correlations are non- significant, but only barely (their 95% CIs are just below zero) and all the CIs overlap, so this is unlikely to reflect meaningful distinctions.

(The corresponding matrix for shared environment correlations is omitted, as there are no significant shared environmental influences on the Bricks measures).

Table S28. Genetic correlations with other measures.

	Mill Hill (verbal)	Raven's Matrices (non-verbal)	*g*
Rotation	0.61<br>(0.24 – 1.00)	0.62<br>(0.41 – 1.00)	0.60<br>(0.36 – 1.00)
Rotation / Visualisation	0.51<br>(0.27 – 0.87)	0.81<br>(0.67 – 1.00)	0.72<br>(0.58 – 1.00)
Visualisation	0.54<br>(0.29 – 0.87)	0.64<br>(0.46 – 0.84)	0.60<br>(0.44 – 0.81)
2D	0.53<br>(0.29 – 0.82)	0.74<br>(0.60 – 0.93)	0.66<br>(0.51 – 0.88)
3D	0.55<br>(0.35 – 0.87)	0.74<br>(0.60 – 0.91)	0.67<br>(0.54 – 0.85)
Overall Bricks	0.51<br>(0.35 – 0.74)	0.77<br>(0.66 – 0.92)	0.69<br>(0.57 – 0.80)

Genetic correlations (95% confidence intervals) with verbal ability, non-verbal ability and *g* (their mean).

Table S29. Trivariate Cholesky decomposition: verbal ability, Rotation, Visualisation.

	**Genetic paths**		
	Verbal	Rotation	Visualisation
1. Verbal	0.46<br>(0.32 – 0.55)		
2. Rotation	0.09<br>(0.01 – 0.21)	0.18<br>(0.03 – 0.35)	
3. Visualisation	0.13<br>(0.04 – 0.27)	0.30<br>(0.06 – 0.41)	*0.00*<br>*(0.00 – 0.00)*

Genetic path estimates (standardised and squared, with 95% confidence intervals) for trivariate ACE Cholesky decomposition. The last row indicates the genetic influences on Visualisation i) shared both with verbal ability (Mill Hill) and with Rotation, ii) shared only with Rotation, and iii) unique to Visualisation. The italicised estimate is non-significant (its CI includes zero).

Table S30. Trivariate Cholesky decomposition: non-verbal ability, Rotation, Visualisation.

	**Genetic paths**		
	Non-verbal	Rotation	Visualisation
1. Non-verbal	0.41<br>(0.27 – 0.54)		
2. Rotation	0.12<br>(0.03 – 0.26)	0.12<br>(0.02 – 0.23)	
3. Visualisation	0.18<br>(0.07 – 0.32)	0.21<br>(0.05 – 0.28)	*0.00*<br>*(0.00 – 0.17)*

Genetic path estimates (standardised and squared, with 95% confidence intervals) for trivariate ACE Cholesky decomposition. The last row indicates the genetic influences on Visualisation i) shared both with non-verbal ability (Raven’s Matrices) and with Rotation, ii) shared only with Rotation, and iii) unique to Visualisation. The italicised estimate is non-significant (its CI includes zero).

Table S31. Trivariate Cholesky decomposition: *g*, Rotation, Visualisation.

	Genetic paths		
	g	Rotation	Visualisation
1. *g*	0.50<br>(0.37 – 0.62)		
2. Rotation	0.10<br>(0.03 – 0.23)	0.17<br>(0.04 – 0.28)	
3. Visualisation	0.16<br>(0.07 – 0.29)	0.24<br>(0.07 – 0.31)	*0.00*<br>*(0.00 – 0.16)*

Genetic path estimates (standardised and squared, with 95% confidence intervals) for trivariate ACE Cholesky decomposition. The last row indicates the genetic influences on Visualisation i) shared both with *g* (the mean of verbal and non-verbal ability) and with Rotation, ii) shared only with Rotation, and iii) unique to Visualisation. The italicised estimate is non-significant (its CI includes zero).

Table S32. Trivariate Cholesky decomposition: verbal ability, 2D, 3D.

	Genetic paths		
	Verbal	2D	3D
1. Verbal	0.47<br>(0.33 – 0.55)		
2. 2D	0.13<br>(0.04 – 0.26)	0.32<br>(0.14 – 0.44)	
3. 3D	0.12<br>(0.05 – 0.25)	0.31<br>(0.13 – 0.39)	*0.00*<br>*(0.00 – 0.08)*

Genetic path estimates (standardised and squared, with 95% confidence intervals) for trivariate ACE Cholesky decomposition. The last row indicates the genetic influences on 3D i) shared both with verbal ability (Mill Hill) and with 2D, ii) shared only with 2D, and iii) unique to 3D. The italicised estimate is non-significant (its CI includes zero).

Table S33. Trivariate Cholesky decomposition: non-verbal ability, 2D, 3D.

	Genetic paths		
	Non-verbal	2D	3D
1. Non-verbal	0.41<br>(0.28 – 0.54)		
2. 2D	0.27<br>(0.14 – 0.42)	0.17<br>(0.06 – 0.25)	
3. 3D	0.20<br>(0.11 – 0.33)	0.16<br>(0.08 – 0.21)	*0.00*<br>*(0.00 – 0.07)*

Genetic path estimates (standardised and squared, with 95% confidence intervals) for trivariate ACE Cholesky decomposition. The last row indicates the genetic influences on 3D i) shared both with non-verbal ability (Raven’s Matrices) and with 2D, ii) shared only with 2D, and iii) unique to 3D. The italicised estimate is non-significant (its CI includes zero).

Table S34. Trivariate Cholesky decomposition: *g*, 2D, 3D.

	Genetic paths		
	g	2D	3D
1. *g*	0.50<br>(0.37 – 0.62)		
2. 2D	0.21<br>(0.11 – 0.34)	0.24<br>(0.11 – 0.32)	
3. 3D	0.19<br>(0.10 – 0.31)	0.21<br>(0.11 – 0.27)	*0.00*<br>*(0.00 – 0.08)*

Genetic path estimates (standardised and squared, with 95% confidence intervals) for trivariate ACE Cholesky decomposition. The last row indicates the genetic influences on 3D i) shared both with *g* (the mean of verbal and non-verbal ability) and with 2D, ii) shared only with 2D, and iii) unique to 3D. The italicised estimate is non-significant (its CI includes zero).

Table S35. Quadrivariate Cholesky decomposition: verbal, non-verbal, Rotation, Visualisation.

	Genetic paths			
	Verbal	Non-verbal	Rotation	Visualisation
1. Verbal	0.45<br>(0.31 – 0.55)			
2. Non-verbal	0.18<br>(0.08 – 0.32)	0.22<br>(0.06 – 0.34)		
3. Rotation	0.08<br>(0.02 – 0.20)	*0.05*<br>*(0.00 – 0.18)*	0.11<br>(0.02 – 0.22)	
4. Visualisation	0.11<br>(0.04 – 0.24)	*0.07*<br>*(0.00 – 0.20)*	0.20<br>(0.03 – 0.26)	*0.00*<br>*(0.00 – 0.16)*

Genetic path estimates (standardised and squared, with 95% confidence intervals) for quadrivariate ACE Cholesky decomposition. The last row indicates the genetic influences on Visualisation i) shared with verbal ability (Mill Hill), non-verbal ability (Raven's Matrices) and Rotation, ii) shared only with non-verbal ability and Rotation (but not verbal ability), iii) shared only with Rotation, and iv) unique to Visualisation. The italicised estimates are non-significant (their CIs include zero).

Table S36. Quadrivariate Cholesky decomposition: verbal, non-verbal, 2D, 3D.

	Genetic paths			
	Verbal	Non-verbal	2D	3D
1. Verbal	0.46<br>(0.32 – 0.55)			
2. Non-verbal	0.18<br>(0.09 – 0.30)	0.23<br>(0.07 – 0.36)		
3. 2D	0.13<br>(0.06 – 0.25)	0.14<br>(0.04 – 0.32)	0.17<br>(0.05 – 0.25)	
4. 3D	0.11<br>(0.05 – 0.16)	0.10<br>(0.02 – 0.25)	0.16<br>(0.05 – 0.21)	*0.00*<br>*(0.00 – 0.07)*

Genetic path estimates (standardised and squared, with 95% confidence intervals) for quadrivariate ACE Cholesky decomposition. The last row indicates the genetic influences on 3D i) shared with verbal ability (Mill Hill), non-verbal ability (Raven's Matrices) and 2D, ii) shared only with non-verbal ability and 2D (but not verbal ability), iii) shared only with 2D, and iv) unique to 3D. The italicised estimate is non-significant (its CI includes zero).

Table S37. Fit statistics: univariate Bricks composite models.

	Model	ep	χ2	df	AIC	Δχ2	Δdf	p
Rotation	Saturated	10	8106.82	2881	2344.82	-	-	-
	ACE	4	8108.17	2887	2334.17	1.35	6	0.97
Rotation / Visualisation	Saturated	10	8079.54	2880	2319.54	-	-	-
	ACE	4	8081.90	2886	2309.90	2.37	6	0.88
Visualisation	Saturated	10	7988.85	2860	2268.85	-	-	-
	ACE	4	7992.22	2866	2260.22	3.36	6	0.76
2D	Saturated	10	8084.56	2902	2280.56	-	-	-
	ACE	4	8086.42	2908	2270.42	1.86	6	0.93
3D	Saturated	10	7936.63	2831	2274.63	-	-	-
	ACE	4	7939.27	2837	2265.27	2.64	6	0.85
Overall Bricks	Saturated	10	7976.85	2889	2198.85	-	-	-
	ACE	4	7979.72	2895	2189.72	2.87	6	0.82

Comparison of univariate ACE models to fully saturated models. ep = estimated parameters; χ2 = -2 log-likelihood; df = degrees of freedom, AIC = Akaike information criterion. The p-values indicate no significant deterioration in fit between the saturated and constrained models (i.e., the ACE models fit well).

Table S38. Fit statistics: bivariate Bricks composite models.

	Model	Ep	χ2	df	AIC	Δχ2	Δdf	p
R, V	Saturated	28	15433.66	5733	3967.66	-	-	-
	ACE	11	15442.00	5750	3942.01	8.34	17	0.96
R, R / V	Saturated	28	15483.91	5753	3977.91	-	-	-
	ACE	11	15499.11	5770	3959.11	15.20	17	0.58
V, R / V	Saturated	28	15233.62	5732	3769.62	-	-	-
	ACE	11	15246.77	5749	3748.77	13.14	17	0.73
2D, 3D	Saturated	28	15041.06	5725	3591.06	-	-	-
	ACE	11	15049.10	5742	3565.10	8.04	17	0.97

Comparison of bivariate ACE models to fully saturated models. Variables were entered in the order specified. R = Rotation; R / V = Rotation and Visualisation; V = Visualisation; ep = estimated parameters; χ2 = -2 log-likelihood; df = degrees of freedom, AIC = Akaike information criterion. The p-values indicate no significant deterioration in fit between the saturated and constrained models (i.e., the ACE models fit well).

Table S39. Fit statistics: trivariate Bricks composite models.

	Model	ep	χ2	df	AIC	Δχ2	Δdf	p
Verbal, R, V	Saturated	54	26544.39	9792	6960.39	-	-	-
	ACE	21	26583.78	9825	6933.78	39.40	33	0.21
Non-verbal, R, V	Saturated	54	25357.27	9549	6259.27	-	-	-
	ACE	21	25392.17	9582	6228.17	34.91	33	0.38
g, R, V	Saturated	54	25353.00	9542	6269.00	-	-	-
	ACE	21	25394.66	9575	6244.67	41.67	33	0.14
Verbal, 2D, 3D	Saturated	54	26118.06	9784	6550.06	-	-	-
	ACE	21	26151.77	9817	6517.77	33.71	33	0.43
Non-verbal, 2D, 3D	Saturated	54	24852.31	9541	5770.31	-	-	-
	ACE	21	24879.46	9574	5731.46	27.15	33	0.75
g, 2D, 3D	Saturated	54	24869.37	9534	5801.37	-	-	-
	ACE	21	24897.55	9567	5763.55	28.18	33	0.71

Comparison of trivariate ACE models to fully saturated models. Variables were entered in the order specified. R = Rotation; R / V = Rotation and Visualisation; V = Visualisation; ep = estimated parameters; χ2 = -2 log-likelihood; df = degrees of freedom, AIC = Akaike information criterion. The p-values indicate no significant deterioration in fit between the saturated and constrained models (i.e., the ACE models fit well).

Table S40. Fit statistics: quadrivariate Bricks composite models.

	Model	ep	χ2	df	AIC	Δχ2	Δdf	p
Verbal, non-verbal,	Saturated	88	36165.57	13600	8965.57	-	-	-
R, V	ACE	34	36237.93	13654	8929.93	72.37	54	0.048
Verbal, non-verbal,	Saturated	88	35650.66	13592	8466.66	-	-	-
2D, 3D	ACE	34	35714.20	13646	8422.21	63.54	54	0.18

Comparison of quadrivariate ACE models to fully saturated models. Variables were entered in the order specified. R = Rotation; R / V = Rotation and Visualisation; V = Visualisation; ep = estimated parameters; χ2 = -2 log-likelihood; df = degrees of freedom, AIC = Akaike information criterion.

N.B. The p-value for the first of these models indicates a significant deterioration in fit between the saturated and constrained model (albeit barely). This may be a chance effect, given the large number of models tested, or the sample size may be underpowered for these larger, more complex models.
